# Helminths of zoonotic importance in Tayassuidae and Suidae in Brazilian Midwest: risks for human and domestic animal health

**DOI:** 10.1017/S0031182025100486

**Published:** 2025-07

**Authors:** Lizandra Fernandes-Silva, Guilherme Oliveira Maia, Bruna Samara Alves-Ribeiro, Zara Mariana Assis-Silva, Georgia Emilly Soares da Costa, João Victor de Oliveira Araújo Alves, Victoria Luiza de Barros Silva, Ellen Amanda Zago, Iago de Sá Moraes, Henrique Trevizoli Ferraz, Marco Antônio de Oliveira Viu, Ísis Assis Braga, Klaus Casaro Saturnino, Richard Campos Pacheco, Edson Moleta Colodel, Thiago Santos Cardoso, Arnaldo Maldonado Junior, Dirceu Guilherme de Souza Ramos

**Affiliations:** 1 Laboratório de Parasitologia e Análises Clínicas Veterinária, Universidade Federal de Jataí, Jataí, GO, Brasil; 2 Laboratório de Anatomia Patológica Veterinária, Universidade Federal de Jataí, Jataí, GO, Brasil; 3 Laboratório de Parasitologia Veterinária e Doenças Parasitárias dos Animais Domésticos e Silvestres, Universidade Federal de Mato Grosso, Cuiabá, MT, Brasil; 4 Instituto de Ciências Agrárias, Universidade Federal de Jataí, Jataí, GO, Brasil; 5 Laboratório de Patologia Veterinária, Universidade Federal de Mato Grosso, Cuiabá, MT, Brasil; 6 Laboratório de Biologia e Parasitologia de Mamíferos Silvestres Reservatórios, Instituto Oswaldo Cruz, Fundação Oswaldo Cruz, Rio de Janeiro, Brasil

**Keywords:** anthropization, invasive fauna, nematodes, wildlife, zoonosis

## Abstract

Common peccaries (*Tayassu pecari*), wild boars (*Sus scrofa*) and collared peccaries (*Dicotyles tajacu*) are species that have gained attention in Brazil because of their close relationship with human beings, their influence on the environment and the parasitic fauna they host, which is common in free-ranging animals. In this study, we obtained the carcasses *S. scrofa* (*n* = 4), *T. pecari* (*n* = 12) and *D. tajacu* (*n* = 1) that were killed by hunting (only wild boars), or by being run over or burned. The animals were necropsied and searched for parasites. The parasites found in the gastrointestinal tract were fixed in ethanol for morphological identification. The parasites identified were *Ascaris suum, Monodontus rarus, Monodontus semicircularis, Strongyloides* spp., *Lagochilascaris minor, Eucyathostomum dentatum, Oligacanthorhynchus major* and *Ascarops strongylina*. In addition to observing the occurrence of different species of parasites in tayassuids and suids, we also aimed to raise awareness among the population about the dangers of consuming these animals, as well as the importance of ecological relationships for the propagation of parasitic fauna. Our results indicate that parasites are host switching, possibly related to the adaptation of *L. minor* and *M. rarus.*

## Graphical abstract

**Figure:**
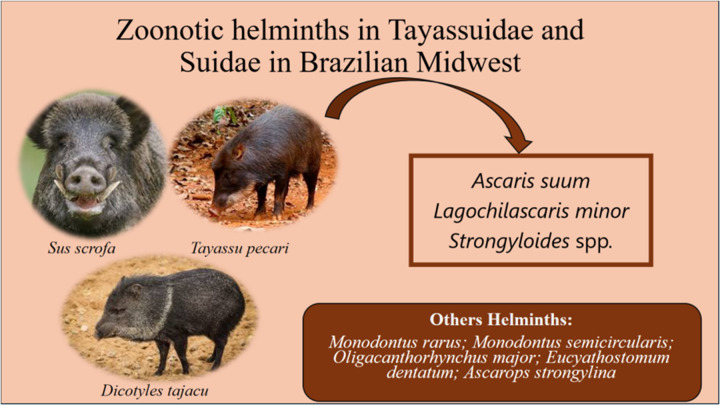

## Introduction

The Brazilian Cerrado, the country’s second-largest biome after the Amazon, is located in the states of Goiás, Distrito Federal, part of Minas Gerais, Rondônia, Mato Grosso, Mato Grosso do Sul, Bahia, Tocantins, Maranhão, Piauí and Pará. This biome occupies around 23% of the national territory and is home to one-third of Brazil’s biodiversity (Santos et al., 2010; Resende, 2012). Emphasizing the importance of conserving this biome, despite the fact that deforestation for agricultural activities, the advancement of cities under native fauna and uncontrolled fires threaten its integrity (Alves-Junior et al., 2013). The same problems affect the Pantanal biome located in Mato Grosso and Mato Grosso do Sul, which contains the largest floodplain in the world (Souza and Souza, 2010). In addition, the abundance of wild species in the Cerrado and Pantanal, for example, is constantly threatened by the increasing number of cattle every year (IBGE, Instituto Brasileiro de Geografia, 2022). Some human activities, such as cattle breeding and hunting, can increase the contact between wild animals, domestic animals and humans. The cattle occupy lands that were once occupied by wild animals; hunters use dogs to sniff and assist during the hunt (Arana et al., 2021). These practices narrow the relationship among wild and domestic animals; these environments can be disturbed when parasites from wild animals infect domestic animals and humans due to the closer relations between these groups (Campos et al., 2017; Souza et al., 2023).

Parasites are commonly found in wild animals, since they are not treated as frequently as domestic animals. Although parasites may be a burden to their individual hosts, they are essential for ecosystem dynamics (Gómez and Nichols, 2013) and play an important role in population control (Leclaire and Faulkner, 2014). Therefore, understanding the dynamics and parasitic constituents of wildlife is a valuable tool for predicting and assessing zoonotic risks (Gałęcki et al., 2015). Despite the knowledge about the relevance of parasites to wildlife dynamics and their implications for human health, there are many gaps to be explored and understood regarding parasitic fauna that infect wild mammals, especially those in tropical climates (Hewavithana et al., 2021).

The order Artiodactyla includes 2 separate families of swine: Tayassuidae and Suidae. The Brazilian Midwest shelters specifically *Tayassu pecari, Dicotyles tajacu* and *Sus scrofa* (wild boar), which have particular prominence related to their interactions with the wild fauna. The peccary (*T. pecari*), native to Brazil, can range from tropical forests to the Cerrado and Caatinga biomes, as well as subtropical areas such as prairies (Castro et al., 2014). The collared peccary (*D. tajacu*), also native to Brazil, is widely distributed from the southern United States to northern Argentina (Pereira-Junior et al., 2016). The wild boar *S. scrofa* arrived in South America as an invasive species, introduced by humans in 1904 for hunting (Piórkowska and Ropka-Molik, 2021; IBAMA, Instituto Brasileiro do Meio Ambiente e dos Recursos Naturais Renováveis, 2025). They are now largely distributed in Brazilian territory, being present in 22 of the 27 states of the federation (Brasil, 2024b). They originate from Eurasia and northern African, and thus, without natural predators that can control their population in South America, their population only increases (Oliveira, 2012).

The expansion of wild boar populations, along with the increasing anthropization of natural habitats, has led to a rise in conflicts or perhaps encounters between humans and both native and domestic animals (Sütő et al., 2020). The contact between wild boars and local species is of zoonotic importance, because wild boars act as hosts for different infectious agents and parasites, such as brucellosis and ascariasis (Dodangeh et al., 2018; Severo et al., 2021). Invasive species represent a risk to local biodiversity by transmitting pathogens to endemic species, a process known as spillover. In contrast, invasive species can act as amplifiers for endemic parasites, promoting the dispersion and increase in abundance of these species in a process called spill-back (Lapera, 2020). The invasive species also compete with local species due to sharing an ecological niche, which occurs mainly due to dietary overlap, leading to competition (Oliveira, 2012; Lima et al., 2022).

The species *S. scrofa, D. tajacu* and *T. pecari* were used in this study. Most of the research involves *S. scrofa* because of its worldwide distribution (Dodangeh et al., 2017; Arana et al., 2021; Belov et al., 2022). The other 2 species are native to America and are being devastated by the presence of wild boars and food competition (Lapera, 2020; Lima et al., 2022). Another problem about study of this species is that scientific names can change during the time which hinders the collection of information, *D. tajacu*, for example, was used to be *Pecari tajacu* (Pereira-Junior et al., 2016). The same helminths described in *D. tajacu* include *Moniezia benedeni, Dirofilaria acutiuscula, Eucyathostomum dentatum, Gongylonema baylisi, Monodontus semicircularis, Nematodirus molini* and *Oesophagostomum dentatum* (Vicente et al., 1997; Pereira-Junior et al., 2016; Justo et al., 2017). In *T. pecari*, the parasites include *Monoecocestus decrescens, Ascaris* sp., *Paragonimus* sp., *Trypanosoma cruzi, Toxoplasma gondii*, and *Oligacanthorhynchus major* (Gomez-Puerta, 2011; Justo et al., 2017; Morais et al., 2017).

The development of research related to wild animals is a challenge in Brazil. Most of the resources are destined for the agriculture sector: being birds, ruminants and fishes the ranking of animals used for teaching and research (CONCEA, Conselho Nacional de Controle de Experimentação Animal, 2024). Working with wild animals comes up against several ethical and legal aspects. Brazilian legislation is quite rigid about the use, manipulation and displacement, even of corpses, being necessary licenses and authorizations specific to study and area (Brasil, Ministério da Justiça, 2008; Nomura, 2012; IBAMA, 2013). Moreover, some species are threatened with extinction or considered vulnerable, which makes it difficult to get permission to manipulate the animal (Gongora et al., 2011; Keuroghlian et al., 2013).

The current ecological scenario in the Brazilian Cerrado, resulting from habitat sharing with wild species, has allowed the entry of new hosts into established parasitic cycles. This study aims to (1) report the occurrence of helminths in wild boars and peccaries in the Cerrado and Pantanal biomes of Brazil’s Midwest region and (2) assess the zoonotic potential of the identified helminth species, helping to address existing knowledge gaps regarding parasitic infections in these animals.

## Materials and methods

### Animals and ethical statement

Four gastrointestinal tracts of *S. scrofa* slaughtered by hunters under the population control measures of the Brazilian Defense Ministry (concession No. 7479935) were analysed. The carcasses of 5 *T. pecari* and 1 *D. tajacu*, which were killed in fires or by being run over on highways in the Midwest, were collected and assessed for parasites, with permission from SISBIO (approval No. 84201-3) between 2021 and 2024. Seven animals were referred by the Wildlife Triage Center after death to necropsy in the laboratory of pathology in Mato Grosso, Brazil.

### Parasitological assessments in intestinal loops

Each animal intestinal loop was inspected during a parasitological necropsy divided in 2 stages: (1) parasites that were visible to the naked eye in the loops and faecal contents were removed and (2) the intestinal contents were washed and filtered through a 0.075-mm sieve, and the retained material was inspected in a Petri dish under a stereoscopic light microscope to obtain any microscopic parasites. The collected parasites were preserved in 70% ethanol (v/v), and the helminths were identified on temporary slides with either 50% glycerol (v/v) or lactophenol solution (Dinâmica) or 90% phenol solution (v/v, Dinâmica) according to the thickness of the helminth (Hoffmann, 1987). The parasites were identified using the taxonomic keys of Vicente et al. (1997), Anderson et al. (2009), Gibbons (2010) and Richardson and Barger (2006). The mean intensity (MI) of each helminth species in each host was calculated, as described by Bush et al. (1997).

### Host–parasite network analysis

Two node centrality statistics (degree and betweenness) were calculated to infer species roles and estimate their importance in the host–parasite network structure (Newman, 2018). Degree centrality refers to the number of direct connections a node has with other nodes in the network and betweenness centrality refers to the number of times a node is on the shortest path between all other nodes, being important to determine how much a species intermediates the connection between all other species (Newman, 2018). These centrality metrics were calculated using the igraph package (Csardi and Nepusz, 2006) in the software R version 4.5.0 (R Core Team, 2025).

A host–parasite interaction network was constructed to describe the interaction patterns between species, considering abundance values of each parasite species in a given host species. The nodes represent the host and parasite species, and the links between the nodes represent the observed interactions between the species. The network representation was built using the software Gephi 0.9.2 (Bastian et al., 2009).

## Results

### Pecarie and wild boars in the Brazilian Cerrado are highly parasitized

**Figure 1.**
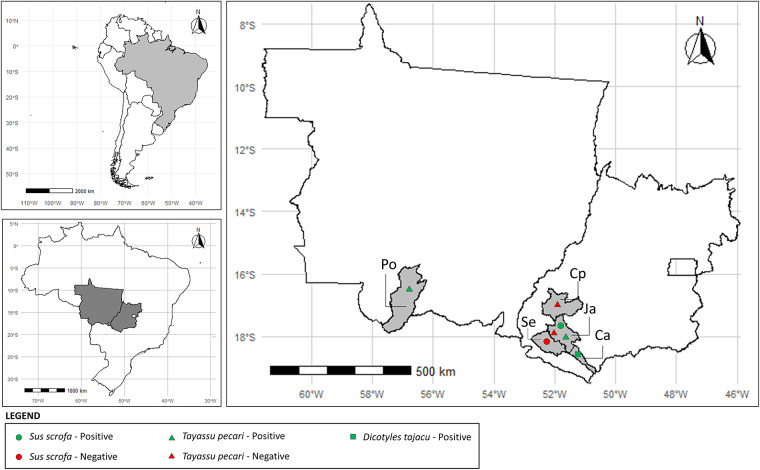
Map of the distribution of Suidae and Tayassuidae positive and negative for helminths in the Central-West of Brazil. Municipalities and states: Po – poconé, Mato Grosso; Se – Serranópolis, Goiás; Cp – Caiapônia, Goiás; Ja – Jataí; Ca – Caçu.

**Figure 2.**
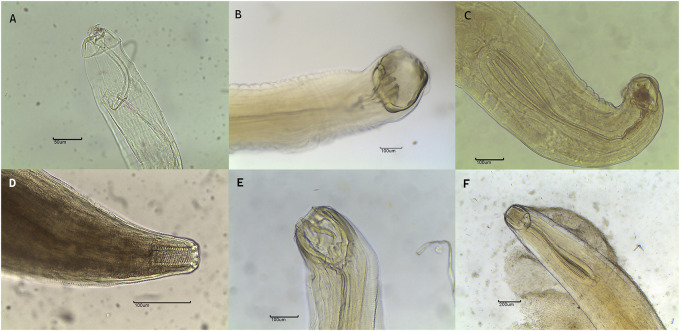
Anterior part of nematodes found in Suidae and Tayassuidae in Central-West Brazil: (A) *Lagochilascaris minor*; (B) *Monodontus semicircularis* showing buccal capsule devoid of teeth or plates but with ventral blades according to Vicente et al. (1997); (C) esophagus of *M. semicircularis*; (D) *Ascarops strongylina*; (E) *Monodontus rarus* with 3 ventral blades according to Vicente et al. (1997); (F) esophagus of *M. rarus.*

The parasitological necropsies revealed the presence of at least 1 parasite species in >70% of the 17 intestinal loops assessed in this study (3 *S. scrofa*, 12 *T. pecari* and 1 *D. tajacu*). Three specimens of *S. scrofa* were positive for *Ascaris suum* (*n* = 2; MI = 4) and *Monodontus rarus* (*n* = 1; MI = 66). Eight *T. pecari* were positive for *A. suum* (*n* = 2; MI = 1), *Lagochilascaris minor* (*n* = 1; MI = 1), *Strongyloides* spp. (*n* = 1; MI = 15), *Monodontus semincircularis* (*n* = 3; MI = 1,37), *Eucyathostomum dentatum* (*n* = 1; MI = 5) and *Oligacanthorhynchus major* (*n* = 3; MI = 2). The distribution of parasitized and non-parasitized animals is shown in Figure 1. The single *D. tajacu* assessed was positive for *Ascarops strongylina* (*n* = 1; MI = 1). Representative examples of morphological characteristics of the specimens: *L. minor, M. semicircularis, A. strongylina* and *M. rarus* are shown in Figure 2.

All parasites were deposited in the helminthological collection of the Universidade Federal de Jataí, Brazil (CHUFJ). The number of samples is presented in Table 1, along with the data from positive animals and co-infections.

**Table 1.** Occurrence of helminths in wild boars (*Sus scrofa*), peccary (*Tayassu pecari*) and collared peccary (*Dicotyles tajacu*) from the Brazilian Cerrado and Pantanal biomes

Haematological collection number	Host	Helminth species	Specimens	Municipality/biome
CHUFJ – 0001	*S. scrofa*	*Ascaris suum*	7	Jataí-GO/Cerrado
CHUFJ – 0002	*S. scrofa*	*A. suum*	1	Jataí-GO/Cerrado
CHUFJ – 0003	*S. scrofa*	*Monodontus rarus*	66	Jataí-GO/Cerrado
CHUFJ – 0004	*T. pecari*	*A. suum*	1	Jataí-GO/Cerrado
CHUFJ – 0005		*Monodontus semicircularis*	1	
CHUFJ – 0006		*Strongyloides* spp.	15	
CHUFJ – 0007	*T. pecari*	*Lagochilascaris minor*	1	Jataí-GO/Cerrado
CHUFJ – 0008	*T. pecari*	*Oligacanthorhynchus major*	3	Jataí-GO/Cerrado
CHUFJ – 0009	*T. pecari*	*M. semicircularis*	1	Jataí-GO/Cerrado
CHUFJ – 0010	*T. pecari*	*M. semicircularis*	2	Jataí-GO/Cerrado
CHUFJ – 0011	*T. pecari*	*O. major*	1	Poconé-MT/Pantanal
CHUFJ – 0012	*T. pecari*	*Eucyathostomum dentatum*	5	Jataí-GO/Cerrado
CHUFJ – 0013	*T. pecari*	*A. suum*	1	Jataí-GO/Cerrado
CHUFJ – 0014		*O. major*	2	
CHUFJ – 0015	*D. tajacu*	*Ascarops strongylina*	1	Caçu–GO/Cerrado

### Parasites sharing

**Figure 3.**
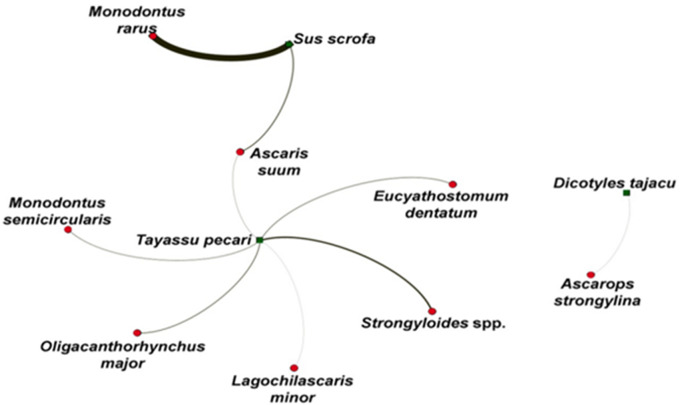
Distribution of parasite species in relation to hosts where 2 clades were identified with the sharing of parasite species between *Tayassu pecari* and *Sus scrofa*, and *Dicotyles tajacu* in a single clade. Thickness of the lines demonstrates a greater abundance of parasites found in the parasite–host interaction.

**Figure 4.**
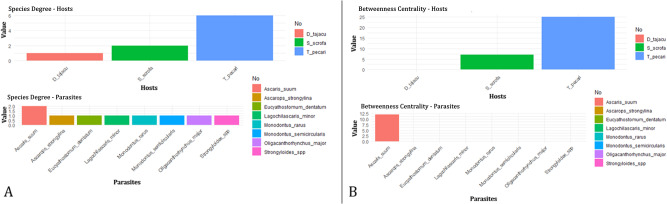
Network centrality. (A) Degree of species by host and by parasites; (B) centrality of species by hosts and by parasites.

We observed a greater diversity of parasite species in *T. pecari*. There is low sharing of parasites between hosts (Figure 3), indicating that parasites tend to be specialized in certain groups of hosts. Thus, there is less sharing of parasites between hosts. It is noted that the only species shared between the host species was *A. suum*. It was observed that the host species with the greatest importance in the network is *T. pecari*, and the parasite species with the greatest importance was *A. suum* (Figure 4). The higher values of network degree and betweenness centrality for *T. pecari* suggest that this species has greater potential to transmit parasites in the network compared to the other species. In turn, the greater degree of network and betweenness-centrality for *A. suum* suggests that this species has greater potential to disperse between hosts.

## Discussion

The geolocalization of wild hosts in this investigation and the parasitic helminths they are infected with are congruent with reports of same infections in domestic animals, namely dogs and cats, thereby reinforcing the zoonotic aspects of these parasites (Souza et al., 2023). In certain regions of Brazil, including in the Cerrado, animals such as pigs and tayassuids are bred and kept close to humans. This close interaction between humans and these animals increases the opportunities for parasite transmission (Andrade et al., 2009).

Free-living animals such as *S. scrofa, T. pecari* and *D. tajacu* are continually exposed to a diverse range of parasites in both natural and anthropogenic environments. The nematode *A. suum* was first reported in Brazilian peccaries in the 1940s, and it is widely distributed throughout South America (Carlos et al., 2008; Quiñajo et al., 2014). It is commonly found in domestic pigs and can damage the economy of pork production systems (Fausto et al., 2015). *Ascaris suum* has been found in *S. scrofa* and *T. pecari* (Belov et al., 2022), and many studies have reported its occurrence in *S. scrofa* worldwide (Hälli et al., 2010; Popiolek et al., 2010; Silva and Muller, 2013; Gassó et al., 2015; Dodangeh et al., 2017). In humans, it can cause symptoms such as a cough, headache, diarrhoea and respiratory discomfort owing to the migration of larvae in the lungs (Silva et al., 2021).

In the present study, *A. suum* was found in 2 different hosts, which suggests its potential dispersion among both native and invasive species (*Sus scrofa*). The increased interactions between these species, humans and domestic animals pose a risk to human health, livestock health and economy, and wildlife ecology. The populations of wild boar increase every year; therefore, the hunting of *S. scrofa* for population control was allowed by law (January legislation; Brasil, 2024a). Such measures should reduce the contact of wild boars with humans, but the effect on parasite transmission must be assessed.

We also identified *Strongyloides* spp. belonging to the superfamily Rhabditoidea. Members of this genus are characteristically host-specific, infecting a diverse range of domestic animal species worldwide (Thamsborg et al., 2016; Jones et al., 2019). However, some species can parasitize humans, non-human primates and wild canids (Thamsborg et al., 2016). In addition, *Strongyloides* spp. affect wild and domestic pigs, as well as Tayassuidae (Nascimento, 2004; Gomes et al., 2005; Brandão et al., 2009; Sampaio et al., 2023). In domestic pigs, *5spp.* cause a decrease in feed conversion, causing losses in pork production (Hale and Marti, 1984). Clinical signs are more frequent in young animals because of their immature immune systems. Notably, parasitized female pigs act as a reservoir, disseminating the parasite to their offspring. Parasitism at the beginning of a piglet’s life predisposes them to other infections (Jacobson, 2022). In domestic pigs, females tend to store the larvae of *Strongyloides* spp. in adipose tissue, eliminating them in colostrum or milk (Thamsborg et al., 2016). Considering the parasite cycle, as known in domestic pigs, and the importance of the females for the dissemination of this parasite to offspring, we suggest that the same can happen in wild swine and Tayassuidae. Although to confirm this hypothesis, further studies must be conducted.

The genus *Eucyathostomum*, identified by Molin in 1861, comprises 3 main species: *E. dentatum, E. longesubulalum* and *E. copulatum.* The specimens of *E. dentatum* used to originally describe the species were coincidentally also isolated from a *D. tajacu*. During the original description of the species, only a small number of gastrointestinal tract parasites were found in the host, and this is similar to the findings of the present study, as only 5 parasites were found in the peccary. Moreover, many studies have reported such infection in Tayassuidae from Brazil and other countries in South America (Nascimento et al., 2005; Pereira-Junior et al., 2016; Quiñajo et al., 2014; Jones et al., 2019). Our results reinforce the notion that *D. tajacu* is a highly adapted host to *E. dentatum*, as previous studies have also identified the same infection in *D. tajacu* in the Amazon and Pantanal regions of Brazil (Nascimento et al., 2005; Pereira-Junior et al., 2016).

*Lagochilascaris minor* is an ascarid that belongs to the genus *Lagochilascaris*, which includes 7 distinct species. Rodents act as intermediary hosts by the development of the parasite in their musculature. This specimen deserves attention because of its host range, which includes humans and carnivores that consume the intermediary hosts (Campos et al., 2017; Trindade et al., 2019; Cupertino et al., 2020). To the best of our knowledge, this is the first report of *L. minor* in *T. pecari*, as it is typically found in felids and canids. Brazil has the highest number of previously described cases of *L. minor* in humans worldwide (Campos et al., 2017). Human lagochilascariasis is an emerging zoonotic disease caused by the consumption of infected game meat with transmission risk supported by the strengthening relationships between wild and domestic animals and humans, owing to habitat sharing (Barrera-Pérez et al., 2012; Falcón-Ordaz et al., 2016; Trindade et al., 2019; Cardoso et al., 2020). Clinical signs of this disease in humans are related to the parasite location. For example, these parasites cause skin lesions that can be confused with abscess. However, erratic migration can lead to their presence in the host nervous system, lungs, sacral region, eyes and oral cavity (Campos et al., 2017).

Contamination by *L. minor* can occur through the consumption of raw or undercooked meat from contaminated animals, such as ungulates, rodents and Tayassuidae, which possibly act as intermediate hosts for this parasite. However, *L. minor* life cycle has not been completely elucidated (Barrera-Pérez et al., 2012).

The increase in cases of *L. minor* infection in different animal species may be related to the migration of prey and predators across different ecosystems, as this allows for the translocation of the parasite, leading to soil contamination in different regions (Campos et al., 2017). Therefore, the *T. pecari* infected in the present study may play a new role as a definite host for this species, a role originally predominating to carnivores (Reis et al., 2011; Falcón-Ordaz et al., 2016; Trindade et al., 2019).

The genus *Monodontus*, first identified in wild boars in 1861 by Molin in Brazil (Travassos, 1937), includes several species, including *M. semicircularis, M. aguiari, M. nefastus* and *M. rarus*, with peccaries, agoutis, tapirs and rodents as their main hosts, respectively. *Monodontus semicircularis* was previously identified in peccaries and *D. tajacu* (Pereira-Junior et al., 2016). This is consistent with the present study, as the original sample used to describe *M. semicircularis* was from a *T. pecari* specimen. *Monodontus rarus* was described in the rodent *Euryzygomatomys guiara*, and this is the first report of *M. rarus* in *S. scrofa*. The literature about this helminth is scarce; therefore, little is known regarding its epidemiology.

*Ascarops strongylina*, belonging to Ascaropsinae, uses the dung beetle as an intermediate host. Reported in *D. tajacu* in Brazil by Nascimento et al. (2005), this species was also found infecting *S. scrofa* (De-La-Muela et al., 2001; Perin et al., 2023), pigs (Sharma et al., 2020), bats (Shimalov, 2021) and rodents (Ganzoring et al., 2003). *Physocephalus sexalatus* is another parasite belonging to the same family, which has already been found in dung beetles, collared peccaries, pigs and wild boars (Samuel and Low, 1970; De-La-Muela et al., 2001; Arriola et al., 2014; Corn et al., 2024). Although most studies have reported *A. strongylina* in *S. scrofa*, none were found in the present study, though we did find the species in *D. tajacu*.

A study in Peru investigated the relationship between these parasites and the occurrence of cysticercosis. Arriola et al. (2014) found that there is a relationship between *A. strongylina* and cysticercosis exposure, likely associated with the consumption of meat, which raises the concern of impact on public health. Only 2 peccaries (8%) presented with co-infection, which is common in wild animals (Karvonen et al., 2019). The presence of the first parasite may predispose the animal to other concomitant infections (Jacobson, 2022), and although we did not find cysticerci, the risk mentioned in the literature should serve as a warning to human populations who may consume the meat of these animals.

We identified *O. major* in 2 peccaries using the description of Brazilian *D. tajacu* described by Machado-Filho (1963). Our study corroborates Gomez-Puerta’s (2011) finding related to the same species of peccaries in Peru.

Destruction of the host’s wild habitat for livestock and crop farming, coupled with population growth, leads to random contact between animal and human populations (Gałęcki et al., 2015), promoting competition for physical and food resources between the Tayassuidae and Suidae. The expansion of the anthropogenic barrier affects parasitic fauna by reducing the number of available hosts. In addition, the reduction in native areas enables more direct contact between species owing to competition for shelter and food, enabling the introduction of new parasites into different species (Weinstein and Lafferty, 2015).

Based on these findings, it is possible to observe that the same parasites did not make up the parasitic fauna of peccaries, possibly because of the adaptation of the parasites due to a reduction in the population of their preferred host. Another hypothesis is related to the reduction in the physical space available for wild animals due to deforestation (Morais et al., 2017), which decreases their geographic range and increases their contact with feces and local waste. Furthermore, it is important to highlight that in some regions of Brazil consumption of meat from wild animals, such as wild boars, peccaries and collared peccaries, is common (Pereira-Junior et al., 2016; Morais et al., 2017), which increases the interaction between parasites from wild animals and humans due to carcass handling and meat consumption, increasing the possibility of being infected with a zoonotic parasite.

Considering the ecological niches, the co-occurrence of taiassuids and suids within the same geographic area provides opportunities for interaction among them. It can be observed that the interaction between taiassuids and suidae occurs due to the occupation of the same geographic area and has a similar diet, as both feed on fruits and vegetables, which can promote the parasitic interaction of helminth communities in both groups (Dodangeh et al., 2017; Lima et al., 2022). Brandão et al. (2009) highlighted that the diet range, together with its opportunistic dietary nature, increased the chances of interaction and contact with different parasites. This may be the reason for the diversity of the helminthological fauna in these animals. Therefore, studies on the parasitic fauna of wild animals, as well as the interactions among the environment, parasites and hosts, must be conducted.

In this study, we observed that some species and genera occurred in all 3 host species, strengthening the hypothesis that the parasitic communities in these groups are shared, and when they occur in the same biome with a high level of anthropization, such as the Brazilian Cerrado, they represent a potential risk for humans and domestic animals, serving as spill-backs. Our conclusions are limited by the number of animals assessed; however, it is important to note that such constraints are inherent to working with wild animals. We only work with occasional samples due to ethical principles, where the animal being studied is more valuable alive than dead for research use, especially *T. pecari*, which is considered a species vulnerable to extinction, and *D. tajacu*, which is already extinct in some areas of its natural occurrence (IUCN, Internation Union for Conservation of Nature and Natural Resources, 2025). Our network analysis showed greater diversity in *T. pecari*, which is expected due to the more expansive nature of this host, coexisting well in anthropized environments and invading crops (IBAMA, Instituto Brasileiro do Meio Ambiente e dos Recursos Naturais Renováveis, 2025), behaviour similar to *S. scrofa* in wild environments, also justifying the presence of a shared species (*A. suum*), as they share habitats and behaviours. This demonstrates that it has characteristics that allow greater ecological flexibility, such as resistance to immunological barriers or the ability to transmit through different routes. Although a single sample of *D. tajacu, A. strongylina*, was not reported in other hosts, even those susceptible to parasitism, demonstrating that this parasite may be more associated with less anthropized locations, since *D. tajacu* avoids locations with a lot of human alteration (IBAMA, Instituto Brasileiro do Meio Ambiente e dos Recursos Naturais Renováveis, 2025).

In conclusion, wild boars and peccaries can be reservoirs and disseminators for different zoonotic parasite species. Notably, this is the first study to report *L. minor* in *T. pecari* and *M. rarus* in *S. scrofa.* Shared ecological niches and dietary similarities allow Tayassuidae and Suidae to share parasitic fauna. Furthermore, the consumption of meat from these animals, together with the aforementioned factors, increases the zoonotic potential of parasites. It is important to constantly monitor and study these animals to evaluate their relationship with the environment and parasites.

## Data Availability

All data generated are included in this manuscript.
